# Editorial: Fruits, vegetables, and biotics for a healthy gut microbiome

**DOI:** 10.3389/fnut.2024.1468453

**Published:** 2024-08-08

**Authors:** Philippe Gérard, Chun Guang Li, Deep Jyoti Bhuyan

**Affiliations:** ^1^Micalis Institute, Université Paris-Saclay, INRAE, AgroParisTech, Jouy-en-Josas, France; ^2^NICM Health Research Institute, Western Sydney University, Penrith, NSW, Australia; ^3^School of Science, Western Sydney University, Penrith, NSW, Australia

**Keywords:** gut microbiota, gut microbiome, postbiotics, prebiotics, probiotics, dietary fiber, fruits and vegetable, gut health

The gut microbiome plays a fundamental role in human health and nutrition, as supported by a growing body of scientific literature. Notably, the gut microbiota and its metabolites have been linked with a variety of chronic conditions, creating new possibilities for gut microbiome modulation-based interventions such as prebiotics, probiotics and postbiotics to maintain health and prevent and treat diseases (1). Fruits and vegetables, with their prebiotic (dietary fiber) and polyphenol content, not only play a crucial role in nutrition but also have the potential to positively influence the gut microbiome, improve gut health, and aid in the prevention and treatment of numerous chronic diseases (2, 3). However, the in-depth mechanisms of action of fruits, vegetables, prebiotics, probiotics and postbiotics on the gut microbiome are still speculative and require further investigation. This Research Topic encompasses eight papers on the regulation of the gut microbiome by fruits, vegetables, and dietary fiber, as well as the underlying molecular mechanisms of action, with a specific focus on developing innovative prebiotic, probiotic, and postbiotic interventions for various health applications.

The study by Procházková et al. examined the impact of a whole-grain-rich diet on colonic fermentation markers and bowel function. Conducted as a randomized controlled cross-over trial, the study involved 50 overweight individuals who alternated between whole-grain and refined-grain diets. Results indicated that the whole-grain diet significantly increased fecal butyrate and caproate levels while also enhancing stool frequency compared to the refined-grain diet. These changes were linked to the activity of butyrate-producing bacteria such as *Faecalibacterium* and *Roseburia*, suggesting that whole grains promoted beneficial fermentation processes in the colon. This study reinforced the idea that dietary fibers in whole grains play a crucial role in maintaining gut health and metabolic balance by modulating the gut microbiome.

In another study, Abuqwider et al. investigated the effects of the probiotic *Limosilactobacillus reuteri* DSM 17938 in both free and microencapsulated forms on rats fed a Western diet. Despite the Western diet's detrimental impact on gut microbiota, supplementation with *L. reuteri* in both forms effectively preserved the integrity of the intestinal barrier. This probiotic intervention prevented the development of inflammation and oxidative stress, common consequences of a Western diet. The findings suggested that *L. reuteri* can mitigate the negative effects of unhealthy diets, and microencapsulation techniques may enhance the viability and efficacy of probiotics, potentially leading to innovative dietary supplements aimed at gut health preservation.

The study by Andújar-Tenorio et al. delved into the prebiotic role of olive oil polyphenols, specifically oleuropein and hydroxytyrosol, in modulating gut microbiota at the strain level. Using a murine model, the researchers examined the impact of high-fat diets enriched with various fats on enterococcal strains. Notably, a diet enriched with extra virgin olive oil (EVOO) led to a more beneficial gut microbiota profile, characterized by less antibiotic resistance and a healthier phenotype. These findings suggested that olive oil polyphenols can positively influence gut microbiota composition, enhancing its adaptability and potentially improving host physiology. This study highlighted the potential of dietary polyphenols in fostering a beneficial gut environment, further supporting the inclusion of EVOO in a balanced diet.

Nishimoto et al. conducted a randomized, double-blind and crossover pilot trial on kale consumption and constipation in Japanese women. The trial included a 4-week dietary intervention period with kale or control food, separated by a 4-week washout period. The authors demonstrated that consuming kale increased stool frequency and altered intestinal microbes and their metabolites, such as the *Eubacterium eligens* group and pimelic acid and *Ruminococcus gnavus* group and morpholine, particularly in constipated women with smaller fecal amounts. These findings indicated the potential health benefit of kale on intestinal function.

Canfora et al. conducted a randomized crossover study on the effect of supplements with a human milk oligosaccharide (2′-fucosyllactose, 2′-FL), compared with placebo, on distal colonic microbial-derived short-chain fatty acids (SCFA) production and metabolic parameters in 10 lean men and nine men with prediabetes and overweight/obesity. The authors showed that supplementation with 2′-FL or 2′-FL plus resistant starch (RS) increased postprandial plasma acetate and fasting H2 excretion in lean men compared to placebo. Postprandial plasma butyrate level was also increased after 2′-FL and 2′-FL with RS treatments, compared to that with placebo with and without RS. These findings suggested the 2′-FL alone or in combination with RS may impact the gut microbiota and have a phenotype-specific metabolic effect.

Eladwy et al. explored the antiproliferative effects of the postbiotic sodium butyrate and quantified its interactions with dexamethasone against AGS gastric adenocarcinoma cells using cellular, molecular and mass spectrometry techniques. Sodium butyrate synergistically interacted with dexamethasone, leading to significant downregulation of the oncogene TNS4 and induction of apoptosis in the gastric adenocarcinoma cells. These findings suggested that postbiotics may enhance the effects of standard chemotherapy; however, further research is needed to understand the fundamental mechanisms comprehensively.

Baek et al. investigated the impact of low (500 mg/kg/day) and high (2,000 mg/kg/day) doses of green banana flour on the gut microbiota of C57BL/6N mice. The research demonstrated that green banana flour exhibited prebiotic activity by significantly altering the gut microbiota after 3 weeks of intervention at both doses. The intervention led to an increase in beneficial gut bacteria such as *Coriobacteriaceae_UCG-002, Turicibacter*, and *Parasutterella*, as well as in biological processes like amino acid and secondary metabolite biosyntheses.

Finally, the review by Campaniello et al. emphasized the promising therapeutic effects of probiotics on various diseases, including colorectal cancer, intestinal disorders, obesity, and diabetes. These effects were primarily attributed to the modulation of gut microbiota and the production of SCFA. The authors noted that most probiotic studies focused on *Lactobacillaceae* and *Bifidobacterium* spp. They also highlighted the need for further research to define efficacy parameters for probiotics and to optimize their dose, storage, duration, and mode of supplementation for better health outcomes.

## Author contributions

PG: Writing – original draft, Writing – review & editing. CL: Writing – original draft, Writing – review & editing. DB: Writing – original draft, Writing – review & editing.
